# Anchor Relative Topology for Sparse Point Correspondence in Sensor-Derived Observations

**DOI:** 10.3390/s26144580

**Published:** 2026-07-20

**Authors:** Xiaoming Wang, Limin Shi

**Affiliations:** 1 School of Science, Shandong Jiaotong University, Jinan 250357, China; wxm_sd@126.com; 2 Institute of Automation, Chinese Academy of Sciences, Beijing 100190, China

**Keywords:** sensor-derived point observations, sparse point correspondence, signal matching, star tracker, retinal imaging, anchor-relative topology, partial assignment

## Abstract

Sparse point correspondence is a recurring signal-processing problem in sensing systems that reduce measurements to compact point observations, including image-control points, detected landmarks, star centroids, and target candidates. Many pipelines first estimate a global transform and only then infer point identities, an order that can be fragile when observations are sparse, partially overlapping, and contaminated by false or missing detections. This paper proposes Anchor Relative Topology (ART), a correspondence-first method that treats anchor-centered relative point layouts as the primary geometric signal. For each candidate anchor pair, ART removes translation by local centering, searches over a bounded rotation variable, evaluates a kernelized topology-consistency score, and recovers a partial one-to-one assignment with explicit unmatched-point handling. Two practical variants are reported: ART-RH increases the anchor-hypothesis budget to improve anchor recall, whereas ART-AD adapts the kernel width and angular grid from local structure. On a 1944-case synthetic benchmark, ART-RH achieves a mean F1 score of 0.7907, compared with 0.6965 for rigid CPD correspondence. Real sensor-derived protocols further show that ART remains effective on retinal image control points and provides useful identity recovery on telemetry-derived star-point cases under strong partial overlap. In the reported synthetic and real sparse-coordinate protocols, the ART variants achieve higher correspondence F1 than the evaluated classical coordinate-only baselines, while retaining an explicit runtime–accuracy trade-off. These results indicate that anchor-relative topology is useful when exact sparse correspondence identities are more important than a single global transform.

## 1. Introduction

Many sensing pipelines reduce raw measurements to sparse point observations before downstream estimation. Examples include control points extracted from retinal images, star centroids used in attitude sensing, visual landmarks, and target candidates in multi-sensor association. In these settings, the downstream task is often not only to fit a transformation but also to recover a discrete partial one-to-one correspondence set between two sparse observations.

Registration-first methods such as ICP, RPM, and CPD remain fast and useful when a global transformation is the main output [1,2,3]. In sparse sensor-derived observations with partial overlap or false detections, however, a plausible global fit can still induce incorrect hard identities. This distinction is important for sensor applications in which the recovered point identities are used by later diagnosis, tracking, pose, or attitude-estimation modules.

The intended operating regime is therefore narrower than general point-cloud registration: ART is useful when the measurements have already been reduced to a small set of coordinates, when some detections are missing or false, and when the downstream module needs point identities rather than only a transformation. Examples include retinal control-point association before image registration, star-centroid association before attitude determination, and sparse target-candidate association before tracking. Conversely, if dense image texture, star catalogs, photometry, or a stable global transform are available and sufficient, a specialized registration or identification pipeline may be preferable. This paper proposes Anchor Relative Topology (ART), a training-free sparse-coordinate correspondence method. ART uses candidate anchors to remove translation, converts the remaining two-dimensional alignment problem into anchor-conditioned one-dimensional rotation searches, and evaluates all hypotheses with a unified kernelized topology-consistency score before a partial assignment step. The method uses only point-coordinate geometry; it does not use image intensities, learned appearance descriptors, star catalogs, celestial angular databases, or attitude priors. ART is therefore positioned as a correspondence-first signal-processing module for sparse sensor outputs and not as a replacement for complete raw-image registration or complete star-tracker pipelines.

The main contributions are as follows: (1) a correspondence-first formulation of sparse sensor-point matching based on kernelized anchor-relative topology; (2) two practical operating variants that expose the roles of anchor recall, kernel tolerance, and angular-search resolution; (3) a representative comparison against descriptor, topology-relaxation, graph-matching, and registration-first baselines with both F1 and runtime reported; and (4) validation on synthetic benchmarks and two sensor-derived sparse-coordinate protocols: retinal image control points and telemetry-derived star-point matching.

## 2. Related Work

Descriptor-based approaches such as Shape Context encode local spatial distributions and then solve an assignment problem [4,5,6]. They are simple and interpretable, but single-point evidence can weaken under repetitive layouts, missing points, or false detections.

Graph and relaxation methods promote pairwise or higher-order consistency among candidate matches [7,8,9]. RRWM, FGM, and IPFP are representative examples [10,11,12]. These methods emphasize correspondence consistency, but their performance remains dependent on candidate generation and compatibility modeling.

Registration-first methods estimate a global transformation and derive correspondences after alignment. ICP, RPM, CPD, and FGR are representative references [1,2,3,13]. RANSAC-style estimators sample minimal geometric hypotheses and verify them by consensus [14], and local-reference-frame methods construct repeatable local coordinate systems before descriptor comparison [15]. TEASER provides certifiable robust registration when putative correspondences are available [16]. These methods are strong when a transformation is the main output or when reliable candidate correspondences are already supplied, but hard identity recovery can still fail if the global fit is attracted by unmatched points or identity-inconsistent alignments. ART differs by accumulating evidence around candidate anchors and then solving a partial correspondence assignment, making it suited to sparse-coordinate sensor protocols with missing points and false detections.

The TEASER comparison is treated as related work rather than as a numerical baseline, because its standard input is a set of putative correspondences for robust registration, whereas the present protocol gives each method only two unlabeled sparse point sets and evaluates exact identity recovery. Adding TEASER would require an additional candidate-correspondence generator, and the result would then measure the generator–TEASER pipeline rather than TEASER alone. We therefore retain CPD, ICP, RPM, Trimmed ICP, Shape Context, and graph-matching baselines as methods that can be run under the same coordinate-only correspondence protocol, and we explicitly avoid claiming superiority over certifiable registration methods in their native setting.

## 3. Materials and Methods

### 3.1. Problem Formulation

Let $X={\left\{x_{i}\right\}}_{i=1}^{N}$ and $Y={\left\{y_{j}\right\}}_{j=1}^{M}$ denote two sparse point sets extracted from two sensor observations. The goal is to recover a partial one-to-one correspondence *C* under noise, outliers, and partial overlap. For an anchor hypothesis (a,b), ART constructs the relative layouts

(1) $$R_{X}\left(a\right)=\left\{x_{i}-x_{a}\right\},\;R_{Y}\left(b\right)=\left\{y_{j}-y_{b}\right\}.$$

This representation removes translation and leaves a local rotation and topology-comparison problem.

The present implementation and experiments address two-dimensional coordinate sets. The same anchor-relative idea can be extended to three-dimensional sensor point clouds by replacing the planar rotation Q(θ) with a three-dimensional rotation R∈SO(3) and evaluating either a sampled rotation set, a branch-and-bound search, or a local optimization initialized by anchor-neighborhood frames. The assignment objective below is dimension independent once distances are computed in the chosen coordinate space. We leave full three-dimensional acceleration and validation for future work because the main computational issue changes from a one-dimensional angular grid to a three-degree-of-freedom rotation search.

**Figure 1:**
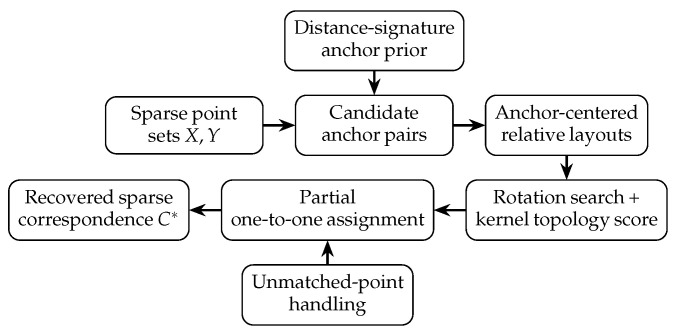
Overview of the ART pipeline. The method treats anchor-relative topology as the sparse geometric signal, evaluates local anchor hypotheses before committing to a global correspondence set, and explicitly allows unmatched points during the final assignment.

Although ART also centers coordinates at an anchor, its objective differs from a local reference frame or a RANSAC modification. A local reference frame normally builds a repeatable coordinate system around a detected keypoint and then compares descriptors. RANSAC samples tentative correspondences and verifies a global model by inlier count. ART instead treats each anchor pair as a hypothesis for a correspondence-first objective: it compares the two full anchor-relative layouts by a soft topology score, selects a bounded rotation, and then solves a gated partial assignment with explicit unmatched decisions. Thus, the anchor is not the final descriptor and not a minimal sample for a global transform; it is a device for translating sparse identity recovery into a set of locally centered topology tests (see Figure 1).

### 3.2. Kernelized Anchor-Relative Scoring

Anchor candidates are generated by a lightweight distance-signature prior before the more expensive topology score is evaluated. Both point sets are first centered and RMS-normalized. For each point, the pairwise distances to all other points are sorted, the first *L* entries are retained, and the retained vector is divided by the mean retained distance over the point set. This gives descriptors $h_{i}^{X}$ and $h_{j}^{Y}$. The anchor-prior matrix is

(2) $$D_{ij}^{A}=\frac{1}{L}{∥h_{i}^{X}-h_{j}^{Y}∥}_{1}.$$

In the default setting L=8, the four lowest-cost target anchors are retained for each source anchor, and entries above the descriptor-cost gate 0.26 are rejected. ART-RH uses a more permissive gate of 0.40 and a larger adaptive candidate budget to improve anchor recall. This prior is intentionally permissive: it does not decide the final correspondence, but it prevents the kernelized topology search from evaluating all NM possible anchors in the practical variants. ART-EX disables this filtering to estimate the ceiling of the topology criterion. For each retained anchor pair, ART searches over rotation angles θ and computes a kernelized compatibility score between the rotated source-relative layout and the target-relative layout:

(3) $$S\left(a,b,\theta \right)=\frac{1}{|R_{X}\left|\right|R_{Y}|}\sum_{u\in R_{X}\left(a\right)}\sum_{v\in R_{Y}\left(b\right)}\exp\left(-\frac{{∥Q\left(\theta \right)u-v∥}^{2}}{\sigma^{2}}\right),$$

where Q(θ) is the rotation matrix, and σ controls the matching tolerance. After the best local orientation is selected, ART builds a local distance matrix and solves a partial assignment with unmatched-point handling.

The Gaussian kernel is used as a soft topological-consistency measure rather than as a probabilistic sensor-noise model. A small σ rewards only nearly coincident relative layouts and can reject true matches under localization noise; a large σ can over-accept false anchors in dense or repetitive layouts. ART-AD therefore estimates σ from the median nearest-neighbor spacing, because this statistic is robust to a small number of outliers and reflects the local coordinate density around the anchor. The clipping limits in Equation (8) prevent very sparse or very dense local neighborhoods from producing unusably wide or narrow kernels. The normalization by $|R_{X}\left|\right|R_{Y}|$ makes S(a,b,θ) an average all-pairs compatibility and keeps scores comparable across anchor hypotheses with different retained relative-set sizes. A normalization by $\min(|R_{X}|,|R_{Y}|)$ would increase scores in imbalanced partial-overlap cases, but it would also inflate anchors surrounded by many false target points. A normalization by the number of plausible matched pairs depends on an additional distance threshold and partly duplicates the later assignment step. For this reason, ART uses the conservative all-pairs average as a screening score and lets the final ranking include the recovered pair count and assignment residual; so, low-overlap hypotheses are not judged by the kernel score alone. This formulation turns two-dimensional alignment into anchor-conditioned one-dimensional rotation searches. The score in Equation (3) filters compatible anchor-centered layouts, while the assignment step imposes a one-to-one structure and rejects unmatched points.

### 3.3. Partial Assignment and Operating Variants

After selecting $\theta^{*}$, ART constructs local distances between $Q\left(\theta^{*}\right)R_{X}\left(a\right)$ and $R_{Y}\left(b\right)$ and solves a partial assignment with unmatched decisions. Candidate sets are ranked by pair count, residual, topology score, and anchor-prior cost.

Let $d_{ij}=∥Q\left(\theta^{*}\right)\left(x_{i}-x_{a}\right)-\left(y_{j}-y_{b}\right)∥$, and let $z_{ij}\in \left\{0,\;1\right\}$ indicate a selected match. Source-unmatched and target-unmatched decisions are represented by $u_{i}$ and $v_{j}$. The final partial assignment can be written as

(4) $$\min_{z,u,v}\;\sum_{i,j}z_{ij}c_{ij}+\lambda_{\mathrm{MD}}\sum_{i}u_{i}+\lambda_{\mathrm{FA}}\sum_{j}v_{j},$$

subject to

(5) $$\sum_{j}z_{ij}+u_{i}=1,\;\sum_{i}z_{ij}+v_{j}=1,\;z_{ij}=0\;\mathrm{if}\;d_{ij}>\delta ,$$

where $c_{ij}=d_{ij}/\delta$ for gated candidate pairs, δ is the assignment gate, $\lambda_{\mathrm{MD}}$ penalizes missed source points, and $\lambda_{\mathrm{FA}}$ penalizes unmatched target points. In the implementation, these two penalties are represented by a dummy cost; setting them finite is what allows partial overlap and false detections instead of forcing a full one-to-one permutation. The reported variants share this objective and differ mainly in anchor-candidate control, kernel-width policy, and angular-grid policy. The retained variants use the same assignment objective. ART-RH raises the anchor budget *K* to improve recall at higher runtime. ART-AD estimates local spacing in $R_{X}\left(a\right)$ and $R_{Y}\left(b\right)$ to set σ(a,b) and adjusts Δθ(a,b) from local distinctiveness. ART-EX is used only to estimate the ceiling of the topology criterion (see Algorithm 1).
**Algorithm 1** Anchor Relative Topology (ART)**Require:** Source points *X*, target points *Y*, descriptor length *L*, anchor gate $\tau_{A}$, target candidates per source *q*, kernel-width policy σ, rotation-grid policy Θ, assignment gate δ, dummy cost λ**Ensure:** Partial one-to-one correspondence set $C^{*}$  1:Center *X* and *Y* and divide each set by its RMS radius.  2:Compute sorted-distance descriptors $h_{i}^{X},h_{j}^{Y}$ and anchor costs $D_{ij}^{A}$ using Equation (2).  3:Retain anchor hypotheses $A=\{\left(i,j\right):D_{ij}^{A}\leq \tau_{A},\;j\in \mathrm{topq}\left(D_{i,:}^{A}\right)\}$; expand the retained set for ART-RH or keep all gated anchors for ART-EX.  4:**for all** (a,b)∈A **do**  5:      Construct relative layouts $R_{X}\left(a\right)={\left\{x_{i}-x_{a}\right\}}_{i\neq a}$ and $R_{Y}\left(b\right)={\left\{y_{j}-y_{b}\right\}}_{j\neq b}$.  6:      Estimate local spacings $s_{X}\left(a\right),s_{Y}\left(b\right)$ and set $\sigma_{ab}$ and $\Theta_{ab}$ from the fixed or adaptive policy.  7:      Evaluate S(a,b,θ) for $\theta \in \Theta_{ab}$ using Equation (3); refine around the best coarse angle when refinement is enabled.  8:      Set $\theta^{*}=\arg\max_{\theta }S\left(a,b,\theta \right)$ and compute gated pair distances after rotating $R_{X}\left(a\right)$.  9:      Solve the partial assignment in Equation (4) with dummy cost λ and gate δ; store pairs, residual, score, and anchor cost.10:**end for**11:**return** $C^{*}$ by lexicographic ranking: larger pair count, smaller residual, larger topology score, and smaller anchor-prior cost.

### 3.4. Complexity

Let *K* be the number of retained anchor hypotheses, *T* the effective number of searched angles including refinement, and N,M the source and target set sizes. The dominant scoring term is O(KTNM), because each retained anchor-angle hypothesis compares the source and target relative layouts. With the final partial assignment included, the main cost is

(6) O(KTNM+KA(N,M)),

where A(N,M) is the cost of the selected assignment solver. In the reported sparse-coordinate setting, the practical scaling is governed primarily by *K*, *T*, and NM. ART-RH is slower, because it raises *K* to improve anchor recall, whereas ART-AD changes σ and the angular grid to balance the tolerance and runtime without an exhaustive anchor search.

### 3.5. Experimental Protocol and Parameter Settings

The evaluation measures exact sparse identity recovery using precision, recall, and F1:

(7) $$P=\frac{TP}{TP+FP},\;R=\frac{TP}{TP+FN},\;F1=\frac{2PR}{P+R}.$$

Registration-first baselines are evaluated after converting their outputs into hard correspondences under the same sparse matching rule. The comparison therefore tests match-identity recovery and not whether these methods are generally effective for image registration. The retained comparison is deliberately coordinate-only: the algorithms receive sparse point locations and do not use raw image intensities, semantic appearance, star catalogs, or downstream attitude-estimation constraints. All methods are evaluated with fixed parameter settings within each benchmark split, without per-case manual tuning.

The retained benchmark suite includes a 1944-case synthetic benchmark with structured and random point families, a 729-case high-outlier stress benchmark, 134 FIRE retinal image control-point pairs [17], and 36 telemetry-derived star-point correspondence cases [18,19]. The star-point benchmark is treated as sparse-coordinate matching from sensor-derived star observations rather than as a complete raw-image star-tracker pipeline. Runtime values are within-study wall-clock measurements from the same implementation environment and are interpreted as relative efficiency indicators. The experiments were run in MATLAB R2025a (version 25.1.0.2943329). Table 1 gives the synthetic benchmark settings needed for reproduction. The main benchmark has 9 point-set families, 3 noise levels, 3 outlier ratios, 3 overlap ratios, and 8 repeats, giving 9×3×3×3×8 = 1944 cases. The stress benchmark keeps the same point families, point count, noise grid, overlap grid, rotation range, translation range, and outlier support, but it uses 3 repeats and outlier ratios 0.25, 0.35, 0.50, giving 9×3×3×3×3 = 729 cases.

All ART parameters are specified after coordinate normalization. The default setting uses descriptor length 8, four target-anchor candidates per source anchor, descriptor-cost gate 0.26, angular search $5^{{}^{\circ}}$ with $\pm 4^{{}^{\circ}}$ refinement at $1^{{}^{\circ}}$, kernel width σ=0.08, dummy cost 0.18, and assignment gate δ=0.16. Here, σ is a normalized spatial tolerance that reflects the localization noise, extraction error, scale normalization, and point density; it is not a direct sensor-accuracy parameter. ART-RH keeps the same kernel and assignment settings but increases the anchor recall by using a robust candidate union with nominal count 6, adaptive maximum 24, target-side candidates, and descriptor-cost gate 0.40. ART-AD estimates the anchor-specific kernel width from local point density,

(8) $$\sigma \left(a,b\right)=\mathrm{clip}\left(0.95\;\mathrm{median}\{s_{X}\left(a\right),s_{Y}\left(b\right)\},\;0.045,\;0.12\right),$$

where $s_{X}\left(a\right)$ and $s_{Y}\left(b\right)$ are median nearest-neighbor spacings in the two anchor-centered layouts. ART-AD also uses adaptive angular steps in $\left[3^{{}^{\circ}},10^{{}^{\circ}}\right]$, $\pm 3^{{}^{\circ}}$ refinement at $1^{{}^{\circ}}$, dummy cost 0.17, assignment gate δ=0.14, and 18 retained hypotheses after coarse screening.

## 4. Results

### 4.1. Overall Synthetic Performance

On the 1944-case overall benchmark, ART-RH ranks first with a mean F1 score of 0.7907, followed by ART-AD at 0.7523 and ART at 0.7516. CPD is the strongest external baseline at 0.6965 and remains substantially faster, defining the central accuracy–efficiency trade-off. Graph-matching baselines are also evaluated on the full benchmark; the best retained graph baseline, IPFP, reaches a 0.2839 mean F1. ART-RH improves the mean F1 over CPD by 0.0942, because its higher anchor budget reduces the early pruning of correct anchors, but the accuracy gain requires evaluating more anchor hypotheses. Table 2 summarizes the full F1-runtime pattern.

ART-RH is therefore the high-recall operating point, ART-AD is the adaptive tolerance and angle-control setting, and default ART is the compact topology setting. The runtime column highlights the main limitation of this family: ART evaluates explicit anchor hypotheses rather than updating a single global registration model.

### 4.2. Structured, Random, and Stress Regimes

The paired statistical summary is reported as per-case F1 differences, 95% confidence intervals for the mean difference, and Cohen’s $d_{z}$ effect size. To make the statistical interpretation independent of a single thresholded significance statement, we report intervals and effect sizes rather than relying on unqualified *p*-values alone. Table 3 shows that ART-RH has a positive mean-F1 advantage over CPD on the full benchmark and on structured prototype families, whereas the random-family subset shows no clear mean-F1 advantage for ART. In the high-outlier stress benchmark, ART-AD has a positive mean-F1 difference over CPD and is the most stable retained ART variant.

An exhaustive-anchor experiment further clarifies the bottleneck. ART-EX reaches mean F1 0.9177 on the focused benchmark, compared with 0.7740 for CPD. Because ART-EX removes anchor filtering, the topology criterion has a high ceiling; the practical challenge is recovering enough correct anchors without an exhaustive cost.

The full graph-matching comparison gives the same qualitative conclusion without relying on a selected subset. IPFP, FGM, and RRWM reach mean F1 scores of 0.2839, 0.2478, and 0.2250, with runtimes around 0.02 s. This should not be read as a general weakness of graph matching; it shows that, under the fixed sparse-coordinate candidate model used here, pairwise relaxation is more sensitive to candidate quality than anchor-centered topology scoring.

The adaptive-parameter benchmark shows that σ mainly controls tolerance to noise and density variation, whereas the angle step controls the speed–resolution trade-off. Adaptive angle stepping reduces the runtime by about 25% on the hardest slice with an almost unchanged F1 (0.4118 versus 0.4117), while the anchor budget *K* remains the dominant recall factor, as shown by ART-RH and ART-EX.

Table 4 separates the main engineering components. Increasing the anchor recall improves the full-benchmark F1 from 0.7516 to 0.7907, and the exhaustive-anchor upper bound reaches 0.9177 on the focused benchmark, showing that anchor recall is the main practical bottleneck, rather than a failure of the topology score. A deliberately aggressive two-stage candidate screen accelerates the ART by 2.43× but reduces the F1 by 0.0512, confirming that early anchor pruning can remove useful hypotheses. Adaptive sigma gives a small overall gain on the focused adaptive benchmark, whereas adaptive angle stepping mainly reduces the runtime. A separate PCA-guided angle ablation was also tested and was not retained because it reduced the full-benchmark F1 from 0.7516 to 0.2538 despite a 1.90× speedup.

**Figure 2:**
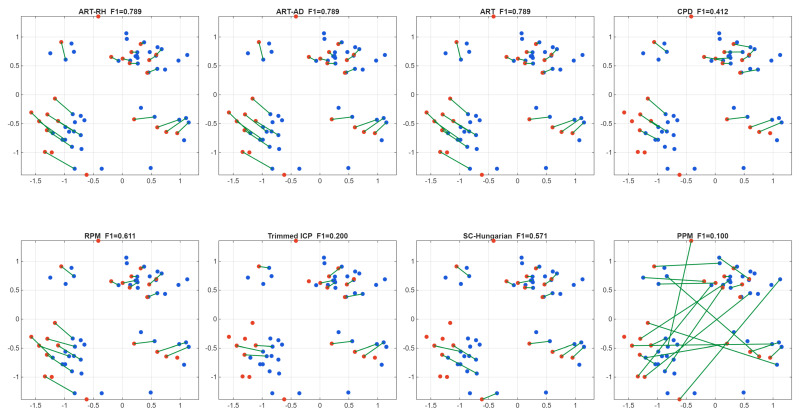
Qualitative correspondence example using the same representative method set as Table 2. Red and blue dots denote source and target points, respectively; green line segments denote predicted correspondences. The selected case is not an all-or-nothing failure example: ART-RH, ART-AD, and ART recover most correspondences, RPM and Shape Context retain partial structure, CPD and Trimmed ICP recover fewer correct identities, and PPM produces several inconsistent links.

Table 5 provides continuous robustness summaries over the tested noise, outlier, and overlap levels. The noise rows show that all ART variants degrade moderately as the localization noise increases to 0.020, while the CPD remains flatter but lower. The outlier rows show the stronger separation: ART-RH is best through 25% outliers in the main benchmark, while ART-AD becomes the most stable ART variant in the supplementary high-outlier benchmark at 50% outliers. The overlap rows show where correspondence-first topology is most useful: ART-RH retains the best F1 at 50% overlap, whereas ART-AD/default ART dominate in cleaner full-overlap cases (Figure 2).

### 4.3. Sensor-Derived Sparse-Coordinate Protocols

The real protocols are complementary sensor-derived correspondence tests. FIRE is introduced first, because its official retinal control points represent an image-sensor setting where registration-first methods are expected to be strong. The telemetry-derived star-point benchmark then changes both the domain and the failure mode: visible point sets vary across frames, many points are unmatched, and identity recovery is required under partial visibility. This progression tests whether the ART behaves as a general sparse sensor-point correspondence method rather than as a dataset-specific retinal matcher.

FIRE provides official retinal image control points. ART and ART-RH reach an F1 of 1.0000, ART-AD reaches 0.9996, Shape Context reaches 0.9941, and PPM reaches 0.9801. With a dataset-scale hard gate for raw pixel coordinates, CPD, RPM, and Trimmed ICP reach 0.9450, 0.1770, and 0.9984. FIRE is therefore a real image-sensor control-point validation where several methods work well, not the strongest differentiating evidence for ART.

**Figure 3:**
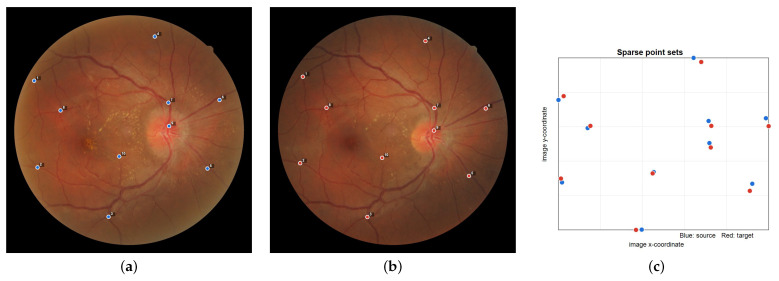
Example origin of the retinal image-sensor sparse-coordinate protocol. The marked FIRE control points are extracted from paired retinal images and then used as sparse-coordinate inputs. Numbers mark the official FIRE control-point identities. The third panel shows the two extracted point sets in a common image-coordinate frame without predicted matching links. ART operates on these point coordinates rather than on image intensities. (**a**) Source image. (**b**) Target image. (**c**) Extracted point sets.

Figure 3 illustrates the FIRE protocol: ART receives extracted image-control-point coordinates rather than raw image intensities.

The star-point benchmark extends the setting beyond retinal imaging to attitude-sensing observations. This experiment uses only the positional coordinates of telemetry-derived star points to test sparse point-set correspondence under partial visibility and many unmatched detections; it is not intended to compete with specialized star-map identification methods that use star catalogs, celestial angular constraints, or attitude-determination models. Under this generic sparse-coordinate protocol, ART-AD reaches an F1 of 0.6104 overall and 0.3838 on the hard subset, ahead of PPM at 0.3491 overall; CPD, RPM, and Trimmed ICP remain low at 0.0242, 0.0131, and 0.0163. A dedicated star-map matching system could use ART as a geometric association core and further combine it with star magnitude, inter-star angular-distance constraints, catalog priors, and temporal attitude information.

**Figure 4:**
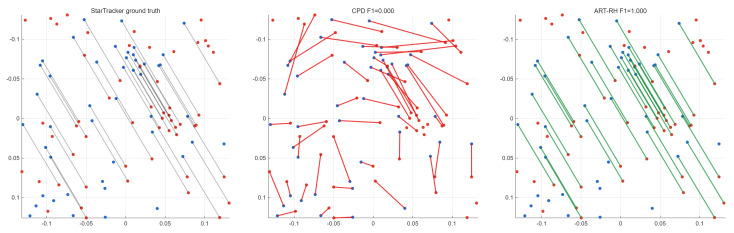
Representative telemetry-derived star-point case under the exact sparse-correspondence protocol. Red and blue dots denote the two point sets; gray, red, and green line segments denote ground-truth, CPD-derived, and ART-RH correspondences, respectively. The left panel shows the ground-truth sparse identities, the middle panel shows CPD-derived links after hard correspondence extraction, and the right panel shows the ART-RH result. CPD produces many identity-inconsistent links in this partial-visibility attitude-sensing setting, whereas ART-RH preserves the correct sparse correspondences.

Across these sensor-derived datasets, descriptor matching is effective when local neighborhoods are distinctive, registration-first methods are strong when the geometry is governed by a stable global transform, and graph-matching relaxations depend heavily on the candidate quality. ART is most useful in the middle regime targeted here: point sets remain sparse and structured enough for relative topology to be informative, but missing or unmatched detections make an early commitment to a single global transform unreliable (Figure 4).

## 5. Discussion

ART should be interpreted as a correspondence-quality method rather than as a speed-oriented registration baseline. Its advantage is strongest when structured relative layouts remain informative but the visible point sets are incomplete. The main limitations are the anchor recall and runtime: if correct anchors are filtered out, the topology criterion cannot express its full strength, and explicit anchor evaluation is heavier than CPD-style global updates. Failure is also more likely when layouts are nearly random, highly symmetric, or so sparsely observed that multiple anchor-centered neighborhoods become indistinguishable.

The runtime trade-off is application dependent. In offline retinal control-point curation or batch telemetry analysis, the additional tens or hundreds of milliseconds per sparse case may be acceptable if it prevents identity errors. In real-time or near-real-time sensing loops, CPD-style registration or a smaller ART candidate budget may be preferable unless the downstream cost of a wrong identity is high. Practical deployments can also combine ART with external priors; star magnitude, inter-star angular distances, catalog constraints, temporal attitude predictions, retinal vessel descriptors, or track-motion priors can reduce the anchor search space before ART evaluates coordinate topology. The application evidence has clear scope limits. The retinal experiment uses sparse image-control-point protocols rather than full raw-image registration, and the star-point experiment uses telemetry-derived coordinate sets rather than an end-to-end centroid extraction and attitude-determination pipeline. Learned graph-matching or transformer-style matchers could improve candidate generation, but they require task-specific training and validation choices outside the present training-free sparse-coordinate setting. In deployment, ART-RH is appropriate when structured ambiguity and partial overlap dominate, ART-AD is preferable under heavier contamination, and registration-first methods remain attractive when runtime is critical and the geometry is close to a globally explainable rigid layout.

For Sensors, the intended role of ART is a sparse-observation association layer between detection and downstream estimation. In image-sensor applications, it can operate on extracted landmarks or control points. In attitude-sensing applications, it can operate on star centroids or telemetry-derived star-point coordinates before final attitude determination. This intermediate positioning explains both the strength and the limitation of the current evidence: the method directly improves sparse correspondence quality, but additional engineering would be required to evaluate a complete sensor-processing chain.

The current scope is also limited to two-dimensional sparse-coordinate experiments. Extending ART to three-dimensional point clouds is conceptually direct but computationally nontrivial, because the angular search space changes from one parameter to SO(3). A practical 3D version would need stronger anchor-frame construction, rotation sampling, or branch-and-bound pruning and should be evaluated on sensor-specific 3D protocols rather than inferred from the present 2D results.

A formal recovery guarantee is not claimed. The empirical behavior suggests the following sufficient conditions for success: at least one correct anchor pair must survive the anchor-prior gate; the correct anchor-relative layout must have a topology-score margin over false anchors under the searched rotation grid; the localization noise must be small relative to the kernel width and assignment gate; and enough overlapping inliers must remain after missing detections. Failures are expected when these conditions break down, especially for random or near-symmetric layouts, very low overlap, dense false detections near true relative positions, or anchor-prior ambiguity that removes all correct anchors. These conditions explain why ART-RH is useful when anchor recall is the bottleneck, why ART-AD is preferable under heavier outlier stress, and why CPD can remain competitive on random point families.

The present work also does not include downstream validation in diagnosis, tracking, pose estimation, or attitude determination. The experiments evaluate the intermediate sparse-correspondence layer only. This is appropriate for isolating the matching problem, but it means that application-level claims should be read as motivation rather than as evidence of downstream system improvement. Future work should integrate ART into complete retinal-registration, star-tracker, and multi-sensor association pipelines and measure task-level errors after the downstream estimator.

## 6. Conclusions

This paper presented ART as a compact signal-processing framework for sparse point correspondence in sensor-derived observations. By centering candidate anchors, scoring kernelized relative topology, and solving partial assignments, ART directly targets the discrete correspondence set. The results show improved match quality in structured and partially overlapping regimes, while confirming that fast registration-first methods remain preferable when runtime or simple global alignment dominates. The ablation and robustness analyses further indicate that anchor recall is the dominant practical factor, kernel width mainly controls tolerance to local density and noise, adaptive angle stepping provides a runtime–resolution trade-off, and unmatched-point penalties are necessary for partial overlap. Future work should focus on learned or physics-informed anchor priors, faster candidate control, three-dimensional rotation search, and integration into complete image-sensor and attitude-sensing pipelines.

## Figures and Tables

**Table 1:** Synthetic benchmark settings. Missing target points are controlled by 1-overlap ratio. Coordinates are normalized before matching.

Setting	1944-Case Main Benchmark	729-Case Stress Benchmark
Point-set families	6 structured + 3 random	same as main
Nominal inlier points	36	36
Repeats	8	3
Noise standard deviation	0.002, 0.008, 0.020	0.002, 0.008, 0.020
Outlier ratio	0.00, 0.10, 0.25	0.25, 0.35, 0.50
Overlap ratio	1.00, 0.75, 0.50	1.00, 0.75, 0.50
Missing target ratio	0.00, 0.25, 0.50	0.00, 0.25, 0.50
Rotation range	$\left[-70^{{}^{\circ}},\;70^{{}^{\circ}}\right]$	$\left[-70^{{}^{\circ}},\;70^{{}^{\circ}}\right]$
Translation range	${\left[-0.35,\;0.35\right]}^{2}$	${\left[-0.35,\;0.35\right]}^{2}$
Outlier support	${\left[-1.4,\;1.4\right]}^{2}$	${\left[-1.4,\;1.4\right]}^{2}$

**Table 2:** Full 1944-case synthetic benchmark. Mean precision, recall, and F1 values of the proposed ART variants are highlighted in bold. Runtime is the mean wall-clock time per case in seconds.

Method	Mean *P*	Mean *R*	Mean F1	Time (s)
ART-RH	**0.7830**	**0.7992**	**0.7907**	0.2711
ART-AD	**0.7502**	**0.7555**	**0.7523**	0.0710
ART	**0.7464**	**0.7578**	**0.7516**	0.1183
Rigid CPD	0.6928	0.7008	0.6965	0.0040
RPM	0.4935	0.4651	0.4753	0.0204
Trimmed ICP	0.4639	0.4526	0.4559	0.0012
Shape Context	0.5622	0.3965	0.4392	0.0078
IPFP	0.2788	0.2903	0.2839	0.0187
FGM	0.2434	0.2533	0.2478	0.0163
PPM	0.2438	0.2219	0.2311	0.2007
RRWM	0.2211	0.2299	0.2250	0.0214

**Table 3:** Paired statistical summary based on per-case F1 differences. ΔF1 is method A minus method B. CI denotes the normal-approximation 95% confidence interval of the paired mean difference, and $d_{z}$ is the paired effect size.

Comparison	Cases	Mean ΔF1	SD (ΔF1)	95% CI	$d_{z}$
ART-RH − CPD, full benchmark	1944	0.0942	0.4742	[0.0731, 0.1153]	0.20
ART-RH − CPD, structured families	1296	0.1483	0.5480	[0.1184, 0.1781]	0.27
ART-RH − CPD, random families	648	−0.0140	0.2384	[−0.0324, 0.0043]	−0.06
ART-AD − CPD, high-outlier stress	729	0.0891	0.4882	[0.0537, 0.1245]	0.18

**Table 4:** Component ablation summary. The full benchmark contains 1944 cases; the exhaustive-anchor row uses a 72-case focused benchmark; the adaptive rows use the focused adaptive-parameter benchmark.

Setting	Component Isolated	Mean F1	Time (s)
ART	default anchor, kernel, angle, assignment	0.7516	0.1183
ART-RH	increased anchor recall	0.7907	0.2711
ART-EX	anchor filtering disabled	0.9177	4.1327
ART two-stage ON	aggressive early anchor screening	0.7004	0.0565
Adaptive sigma only	density-based kernel width	0.5827	0.2347
Adaptive angle only	adaptive angular resolution	0.5772	0.1709
Fixed ART in adaptive benchmark	fixed reference for previous two rows	0.5813	0.2310

**Table 5:** Robustness curves summarized as mean F1 under individual condition levels. Noise, outlier, and overlap rows are marginal summaries over the remaining benchmark factors; the 0.35 and 0.50 outlier rows are from the supplementary 729-case high-outlier stress benchmark.

Factor	Level	ART-RH	ART-AD	ART	CPD
Noise $\sigma_{n}$	0.002	0.7966	0.7683	0.7631	0.6993
Noise $\sigma_{n}$	0.008	0.8018	0.7624	0.7616	0.6953
Noise $\sigma_{n}$	0.020	0.7738	0.7263	0.7303	0.6950
Outlier ratio	0.00	0.8783	0.8367	0.8423	0.7582
Outlier ratio	0.10	0.8179	0.7612	0.7690	0.7201
Outlier ratio	0.25	0.6760	0.6591	0.6436	0.6114
Outlier ratio	0.35	0.6358	0.6456	0.6544	0.5355
Outlier ratio	0.50	0.5214	0.5844	0.5567	0.4489
Overlap ratio	0.50	0.6822	0.5899	0.5918	0.6036
Overlap ratio	0.75	0.7977	0.7500	0.7603	0.7271
Overlap ratio	1.00	0.8923	0.9172	0.9028	0.7589

## Data Availability

Benchmark scripts and processed sparse-coordinate protocols will be made available upon publication, subject to the licenses of the original datasets.

## Abbreviations

The following abbreviations are used in this manuscript:

ART	Anchor Relative Topology
AD	Adaptive Density
CPD	Coherent Point Drift
F1	Harmonic mean of precision and recall
FIRE	Fundus Image Registration Dataset
FGM	Factorized Graph Matching
ICP	Iterative Closest Point
IPFP	Integer Projected Fixed Point
PPM	Point-pattern Matching
RH	Robust high-recall hypothesis search
RPM	Robust Point Matching
RRWM	Reweighted Random Walks Matching
SC	Shape Context

## References

[1] Besl P.J., McKay N.D. (1992). A method for registration of 3-D shapes. IEEE Trans. Pattern Anal. Mach. Intell..

[2] Chui H., Rangarajan A. (2003). A new point matching algorithm for non-rigid registration. Comput. Vis. Image Underst..

[3] Myronenko A., Song X. (2010). Point set registration: Coherent Point Drift. IEEE Trans. Pattern Anal. Mach. Intell..

[4] Belongie S., Malik J., Puzicha J. (2002). Shape matching and object recognition using shape contexts. IEEE Trans. Pattern Anal. Mach. Intell..

[5] Kuhn H.W. (1955). The Hungarian method for the assignment problem. Nav. Res. Logist. Q..

[6] Munkres J. (1957). Algorithms for the assignment and transportation problems. J. Soc. Ind. Appl. Math..

[7] Scott G.L., Longuet-Higgins H.C. (1991). An algorithm for associating the features of two images. Proc. R. Soc. Lond. Ser. B.

[8] Leordeanu M., Hebert M. A spectral technique for correspondence problems using pairwise constraints. Proceedings of the IEEE International Conference on Computer Vision.

[9] Deng W., Zou H., Guo F., Lei L., Zhou S. (2018). Point-pattern matching based on point pair local topology and probabilistic relaxation labeling. Vis. Comput..

[10] Cho M., Lee J., Lee K.M. (2010). Reweighted random walks for graph matching. Proceedings of the European Conference on Computer Vision.

[11] Zhou F., De la Torre F. (2012). Factorized graph matching. Proceedings of the IEEE Conference on Computer Vision and Pattern Recognition.

[12] Leordeanu M., Hebert M., Sukthankar R. (2009). An integer projected fixed point method for graph matching and MAP inference. Proceedings of the Advances in Neural Information Processing Systems.

[13] Zhou Q.Y., Park J., Koltun V. (2016). Fast global registration. Proceedings of the European Conference on Computer Vision.

[14] Fischler M.A., Bolles R.C. (1981). Random sample consensus: A paradigm for model fitting with applications to image analysis and automated cartography. Commun. ACM.

[15] Tombari F., Salti S., Di Stefano L. (2010). Unique signatures of histograms for local surface description. Proceedings of the European Conference on Computer Vision.

[16] Yang H., Shi J., Carlone L. (2021). TEASER: Fast and certifiable point cloud registration. IEEE Trans. Robot..

[17] Hernandez-Matas C., Zabulis X., Triantafyllou A., Anyfanti P., Douma S., Argyros A.A. (2017). FIRE: Fundus Image Registration dataset. J. Model. Ophthalmol..

[18] Rijlaarsdam D., Yous H., Byrne J., Oddenino D., Furano G., Moloney D. (2020). A survey of lost-in-space star identification algorithms since 2009. Sensors.

[19] Hu D. (2025). Star tracker attitude data. Zenodo.

